# Validity and reliability of the 9-item Shared Decision Making Questionnaire (SDM-Q-9) in a national survey in Hungary

**DOI:** 10.1007/s10198-019-01061-2

**Published:** 2019-05-20

**Authors:** Fanni Rencz, Béla Tamási, Valentin Brodszky, László Gulácsi, Miklós Weszl, Márta Péntek

**Affiliations:** 1 Department of Health Economics, Corvinus University of Budapest, Fővám tér 8, Budapest, 1093 Hungary; 2 Premium Postdoctoral Research Programme, Hungarian Academy of Sciences, Nádor u. 7, Budapest, 1051 Hungary; 3 Department of Dermatology, Venereology and Dermatooncology, Faculty of Medicine, Semmelweis University, Mária u. 41, Budapest, 1085 Hungary

**Keywords:** Shared decision-making, SDM-Q-9, Primary care, Specialised care, Psychometrics, I10

## Abstract

**Background:**

The nine-item Shared Decision Making Questionnaire (SDM-Q-9) is one of the most frequently applied instruments for assessing patients’ involvement in medical decision-making. Our objectives were to develop a Hungarian version of SDM-Q-9, to evaluate its psychometric properties and to compare its performance between primary and specialised care settings.

**Methods:**

In 2019, a sample of adults (*n* = 537) representative of the Hungarian general population in terms of age, gender and geographic region completed an online survey with respect to a recent health-related decision. Outcome measures included SDM-Q-9 and Control Preferences Scale-post (CPS_post_). Item characteristics, internal consistency reliability and the factor structure of SDM-Q-9 were determined.

**Results:**

The overall ceiling and floor effects for SDM-Q-9 total scores were 12.3% and 2.2%, respectively. An excellent internal consistency reliability (Cronbach’s alpha 0.925) was demonstrated. Exploratory factor analysis resulted in a one-factor model explaining 63.5% of the variance of SDM-Q-9. A confirmatory factor analysis supported the acceptability of this model. Known-groups validity was confirmed with CPS_post_ categories; mean SDM-Q-9 total scores were higher in the ‘Shared decision’ category (72.6) compared to both ‘Physician decided’ (55.1, *p* = 0.0002) and ‘Patient decided’ (57.2, *p* = 0.0086) categories. In most aspects of validity and reliability, there was no statistically significant difference between primary and specialised care.

**Conclusions:**

The overall good measurement properties of the Hungarian SDM-Q-9 make the questionnaire suitable for use in both primary and specialised care settings. SDM-Q-9 may be useful for health policies targeting the implementation of shared decision-making and aiming to improve efficiency and quality of care in Hungary.

## Introduction

In many countries, increasing patient engagement in healthcare is advocated by health policy [1]. Shared decision-making (SDM) is defined as a process by which health-related decisions are made jointly by the physician and the patient. Steps of SDM include an open communication about a decision that needs to be made, informing the patient about the choices available, eliciting patients’ preferences regarding the decision, providing help for the patient to weigh the risks versus benefits and ultimately supporting the patient to play an active role in making the decision [2]. SDM has the potential to provide numerous benefits including increased patient knowledge, improved health outcomes, reductions in costs and greater alignment of care with patients’ values [3–7]. Patient participation in medical decision-making is increasingly recognised as a tool to reduce health inequalities and a quality indicator of healthcare systems [8, 9]. While in many European countries SDM has become a health policy priority in the past two decades, the literature about the involvement of patients in medical decisions in Hungary is scarce [10–12].

A recent systematic literature review identified 16 existing patient questionnaires pertaining to SDM [13]. The nine-item Shared Decision Making Questionnaire is one of the most frequently applied instruments for assessing the extent to which clinicians involve patients in decision-making. It consists of a patient (SDM-Q-9) and a physician (SDM-Q-Doc) version that allow to assess the patients’ involvement in decision-making from two perspectives [14, 15]. It has been widely used in various clinical settings including primary and specialised care along with clinical trials and national surveys [16, 17]. Studies have shown that SDM-Q-9 is a useful measure in a number areas of medicine, such as anaesthesiology [18], cardiovascular diseases [19, 20], dermatology [21], mental illnesses [22–24], oncology [25–27], otolaryngology [28], and traumatology [29]. Since its development in 2009 it has been translated to over 20 languages. It demonstrated a good internal consistency and construct validity in numerous studies [14, 24, 30–34]. Recent research, however, indicates that still there is a clear need for quality improvement in validation studies, for example, in terms of sample sizes, methodological quality, finding ways to quantify known-groups validity and to compare its measurement properties across different levels of healthcare system [13, 30].

To date, no Hungarian version of SDM-Q-9 has been available. Therefore, the primary objective of the present study was to develop a Hungarian version of SDM-Q-9 and to evaluate its psychometric properties as a part of a large national survey on SDM practices in Hungary. A set of measurement properties of the instrument is analysed including internal consistency reliability, factor structure and known-groups validity. Our secondary aim was to compare the performance of SDM-Q-9 in primary and specialised care.

## Methods

### Study design and participants

In early 2019, an internet-based questionnaire was administered to a national sample of adults in Hungary. Recruitment for the study was conducted through a specialised survey company (Big Data Scientist Ltd.). Volunteers enlisted with this company were invited to participate in the study. The study invitation was sent via the company to the selected volunteers. Participation was anonymous and no compensation of any kind was provided to the respondents. The study received approval from the National Scientific and Ethical Committee (reference no. 47654-2/2018/EKU) prior to data collection. Inclusion criteria to the study were (i) aged ≥ 18 years and (ii) signed an informed consent form.

A stratified random sampling was applied to recruit 1000 respondents stratified on age, gender, education level, place of residence and geographic region that reflects the composition of the Hungarian general population according to the Hungarian Central Statistical Office (KSH) [35]. Given the lower use of internet among individuals aged ≥ 65 [36], the sampling aimed to reflect the distribution of each stratum between the age of 18 and 65, but not in the over-65 age groups. Data of participants reported having a consultation with a physician within the past 6 months for a health-related decision on any levels of healthcare (primary or specialised care) were considered. The recall period was set at the preceding 6 months, because it was considered short enough to remember a consultation with a physician, but long enough not to exclude a large number of respondents. This is consistent with large national surveys on SDM in other countries that used various time frames ranging from 3 to 12 months [17, 37–39].

### The questionnaire

The questionnaire was a part of a longer survey covering many topics asked in three separate modules (e.g. electronic health literacy, SDM and patient-reported experience measures). In the SDM module of the questionnaire, participants were first asked whether they had a health-related decision in a consultation with a physician within the past 6 months. Respondents were also questioned about the level of care (i.e. primary or specialised) with reference to the decision made. Then, they completed a Control Preferences Scale-post (CPS_post_) and SDM-Q-9. Demographics and participants’ general health status were also recorded. The Minimum European Health Module was administered to assess self-perceived health, chronic morbidity and activity limitations [40, 41]. All questions of the survey were set at mandatory, so respondents could not proceed to the next question without answering the previous one.

### Measures

#### SDM-Q-9

The SDM-Q-9 is self-reported questionnaire designed to assess patients’ views on SDM occurred in a consultation with a healthcare provider [14]. It contains two open-ended questions [‘Please indicate which health complaint/problem/illness the consultation was about’ and ‘Please indicate which decision was made’] followed by nine closed questions. Each closed question is represented by a statement featuring various aspects of SDM, rated on a 6-point balanced scale ranging from 0 (= ‘completely disagree’) to 5 (= ‘completely agree’). The total score, calculated by summing the score of the nine items, is expressed on a scale ranging between 0 and 45, where a higher score represents a greater level of perceived SDM. Following earlier studies, we rescaled the raw total scores to a 0–100 range [14, 30]. Completion time of SDM-Q-9 was recorded for all participants.

#### Translation of the questionnaire

The permission to translate and use SDM-Q-9 was obtained from the developer core team of the questionnaire (University Medical Center Hamburg-Eppendorf, Germany). The translation and cross-cultural adaptation process followed the guidelines of Beaton et al. [42]. Two Hungarian researchers independently translated the original German version of SDM-Q-9 into Hungarian. The two translations have been harmonised through discussion until the first consensus version was agreed upon. The consensus version has been back-translated to German by a third independent translator blind to the original version. The back translation was sent to the developers of the questionnaire who commented on that. This led to certain changes in the first consensus version to reach the second consensus version, approved by the developer team. Similarly to the English translation of SDM-Q-9, we preferred to use a passive voice for the second open-ended question ‘What decision was made?’ (Hungarian: ‘Milyen döntést hoztak?’). Moreover, we decided to use ‘told’ (Hungarian: ‘elmondta’) as the translation of the German verb ‘mitgeteilt’ (English: ‘informed’ or ‘communicated’) often has a negative connotation in Hungarian (‘közölte’). A cognitive debriefing interview of the second consensus version was carried out with five individuals. Based on these interviews, no modification was required to the second consensus version, which resulted in the final Hungarian version of SDM-Q-9. The SDM-Q-Doc has also been translated as a part of the translation process; however, it was not used in the present study. The SDM-Q-9 and SDM-Q-Doc are complement to one another but can be validated separately [15, 43].

#### Content coding of decisions

Responses on the two open-ended questions of SDM-Q-9 were analysed using a content analysis framework [44]. Analyst triangulation was used to ensure credibility of the results [45]. The categories were proposed by the lead researcher (F.R.), following a discussion with the team members and bearing in mind comparability with previous large national surveys on shared decision-making in other countries [17, 37, 46]. Responses were coded according to categories by two researchers independently (F.R. and B.T.). Disagreements were resolved through discussion with a third researcher (M.P.) If a respondent indicated several reasons for the consultation, only those that were associated with a clear decision were included. Respondents indicating an unspecified illness/symptom/problem for the reason of consultation but providing a clear specification of the type of decision made were included in the analysis (e.g. reason for visit: ‘bleeding’, type of decision: ‘surgery’).

#### CPS_post_

The questionnaire involved a modified version of Control Preferences Scale (CPS) [47], the CPS_post_ [48] to assess known-groups validity of SDM-Q-9. The CPS_post_ is a single-item measure to evaluate patients’ perceived participation in health-related decisions. Evidence suggests that the CPS_post_ is a valid and reliable measure of patient involvement in medical decisions [30, 48, 49]. It has five response options describing the role of the patient in the physician–patient-interaction: 1 (= ‘I made my decision alone’), 2 (= ‘I made my decision alone considering what my doctor said’), 3 (= ‘I shared the decision with my doctor’), 4 (= ‘My doctor decided considering my preferences’), and 5 (= ‘My doctor made the decision’).

### Statistical analyses

The following exclusion criteria were specified a priori based on the two open-ended questions of SDM-Q-9:

1. The decision was made during a visit at the dentist, psychologist, nutritionist, physiotherapist or veterinarian.
2. The respondent provided nonsensical responses to any of the open-ended questions.

Descriptive characteristics of the sample were computed. Item analysis of SDM-Q-9 questionnaire involved the estimation of the distribution of responses to each item, item difficulties, discrimination and internal consistency. Ceiling and floor effects, expressed as the proportion of ‘completely agree’ and ‘completely disagree’ responses per item, were considered to be present if ≥ 15% of respondents achieved the highest or lowest possible score, respectively [50]. The difference in the presence of ceiling and floor effects between the primary and specialised care sample was tested using Fisher’s exact test. Item difficulties were determined by calculating the mean total score of each item. In line with former validation studies, a mean score below the midpoint (2.5 on a scale ranging between 0 and 5) was interpreted as a generally difficult aspect of SDM in a consultation [30]. Perceived difficulty and SDM-Q-9 total scores between primary and specialised care were compared using Student’s *t* test.

Discrimination (i.e. how efficient the items individually contribute to the scale) was assessed by computing corrected item-total correlations and the value of Cronbach’s alpha (*α*) if the item was deleted. Internal consistency reliability of the SDM-Q-9 scale as a whole was assessed using Cronbach’s α [51]. Internal consistency was considered good if 0.8 ≤ *α* < 0.9 and excellent if *α* > 0.9 [52]. The Cronbach’s *α* values of primary and specialised care subsamples were compared using Feldt’s test [53].

Construct validity of SDM-Q-9 was examined by exploratory factor analysis (EFA) and confirmatory factor analysis (CFA). Regarding EFA, the eigenvalue > 1 rule and the scree plot were used to determine the number of factors. The appropriateness of the factor model was assessed by the Kaiser–Meyer–Olkin (KMO) measure of sampling adequacy [54] and the significance of the Bartlett’s test of sphericity. The recommended value for the KMO was ≥ 0.5 [55]. The quality of items was judged based on estimating factor loadings, inter-item correlations and communalities (*h*^2^). Factor loadings were interpreted as acceptable if ≥ 0.3, practically significant if ≥ 0.5 and indicative of a well-defined structure if ≥ 0.7. The desired value for inter-item correlation coefficients was being lower than 0.85 [56]. A *h*^2^ was deemed acceptable if > 0.5 [55].

In the second stage of factor analyses, a CFA was conducted. Following the Dutch and Spanish validation studies, four single-factor model specifications were tested: all nine items (Model 1); excluding item 1 (Model 2), excluding item 9 (Model 3) and excluding items 1 and 9 (Model 4) [30, 31]. Due to the non-normal distribution of data, we used both maximum likelihood and robust estimators (Satorra-Bentler) [57]. Multiple criteria were employed to assess goodness-of-fit of the models: Chi-square statistic (*χ*^2^), comparative fit index (CFI), root mean square error of approximation (RMSEA) and standardized root mean square residual (SRMR). The desired threshold values were > 0.90 for CFI and ≤ 0.8 for both RMSEA and SRMR [58].

Known-groups validity of the SDM-Q-9 with CPS_post_ was evaluated by comparing the differences in SDM-Q-9 total scores across the five categories of CPS_post_. Analysis of variance (ANOVA) and Games–Howell post hoc test were employed. We hypostatised the highest mean SDM-Q-9 scores on the CPS_post_ for the ‘Shared decision’ category.

A *p* value of < 0.05 was considered statistically significant for all analyses. CFA was carried out using Stata 14 (College Station, TX: StataCorp LP.), the Feldt’s test was carried out in R using ‘cocron’ command [59] and all other statistical analyses were performed using SPSS 25.0 (Armonk, NY: IBM Corp.)

## Results

### Sample characteristics

**Fig. 1:**
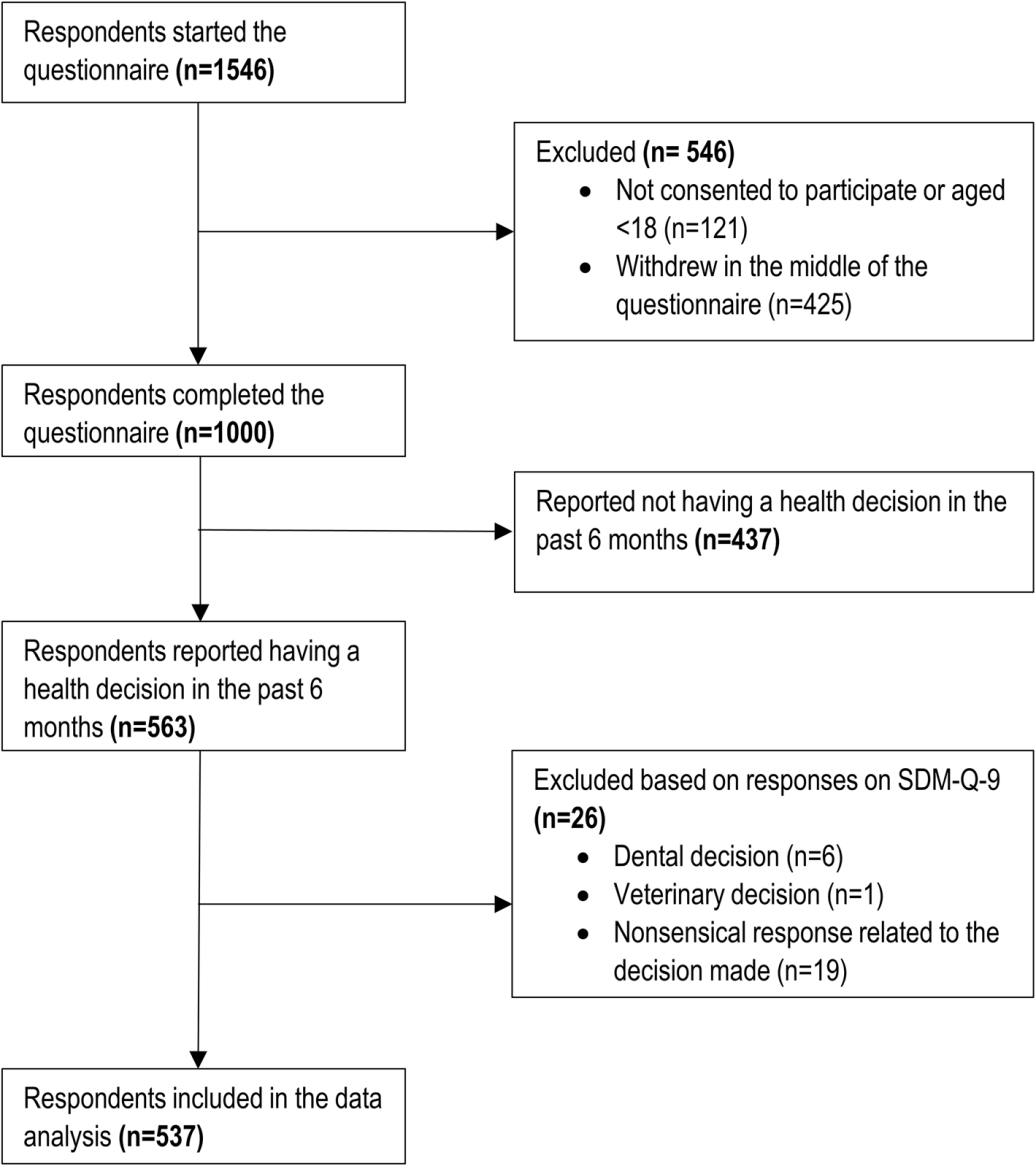
Study flow chart. *SDM-Q-9* 9-item Shared Decision Making Questionnaire

Out of the 1546 respondents who started the online questionnaire (consisting on three modules, as described above), a total of 546 were excluded. Out of these, 121 participants declined to consent to the study or aged <18 years, and further 425 decided to withdraw in the middle of the survey. The valid sample consisted of 1000 respondents, 563 of whom reported having a health-related decision in the past 6 months. A total of 26 respondents were excluded according to the exclusion criteria related to the quality of responses on SDM-Q-9 (Fig. 1). The most common reason for exclusion was providing a nonsensical response to the open-ended questions (e.g. ‘I don’t know’ or ‘this is a private matter’). Thus, data of 537 respondents were analysed in the present study.

Sociodemographic characteristics and general health status of the participants are presented in Table 1. Mean age was 49.4 (SD 18.0, range 18–90) years. The sample well represented the Hungarian general population for gender, age (except for the over-65 age groups), place of living and geographical region. Higher educated respondents were somewhat overrepresented, and respondents with lower educational background were underrepresented in the sample. The presence of chronic morbidities and activity limitations were more prevalent among respondents compared with the general population. Of the 537 participants included, responses of 211 (39.3%) and 320 (59.6%) referred to a decision made in primary and specialised care settings, respectively, while 6 (0.9%) respondents indicated other level of care.

**Table 1:** Characteristics of the study population (*n* = 537)

Variables	*n*	%	Hungarian general population (%) [35, 60]	Proportional difference (%)
Gender				
Female	290	54	53.1	0.9
Male	247	46	46.9	− 0.9
Age (years)				
18–24	50	9.3	10.0	− 0.7
25–34	89	16.6	15.2	1.4
35–44	92	17.1	19.5	− 2.4
45–54	64	11.9	16.0	− 4.1
55–64	89	16.6	16.8	− 0.2
65–74	126	23.5	13.0	10.5
75 +	27	5	9.5	− 4.5
Highest level of education				
Primary school	83	15.5	23.8	− 8.3
Secondary school	281	52.3	55.0	− 2.7
College/university	173	32.2	21.2	11.0
Place of residence				
Capital	110	20.5	17.9	2.6
Other town	305	56.8	52.6	4.2
Village	122	22.7	29.5	− 6.8
Region^a^				
Northern Hungary	69	12.8	11.7	1.1
Northern Great Plain	68	12.7	14.9	− 2.2
Southern Great Plain	62	11.5	13.0	− 1.5
Central Hungary	183	34.1	30.4	3.7
Central Transdanubia	58	10.8	10.8	0.0
Western Transdanubia	39	7.3	10.1	− 2.8
Southern Transdanubia	58	10.8	9.2	1.6
Minimum European Health Module				
Self-perceived health				
Very good	35	6.5	19	− 12.5
Good	229	42.6	42	0.6
Fair	213	39.7	28	11.7
Bad	56	10.4	8	2.4
Very bad	4	0.7	3	− 2.3
Chronic morbidity^a,b^				
Yes	335	62.4	45	17.4
No	140	26.1	55	− 28.9
Activity limitations (GALI)^a,c^				
Not limited at all	240	44.7	70.8	− 26.1
Limited but not severely	220	41.0	20.0	21.0
Severely limited	48	8.9	9.2	− 0.3

*GALI* global activity limitation indicator

^a^General population percentages are reported for the 15 + population

^b^*n* = 62 (11.5%) did not know or refused to answer

^c^*n* = 29 (5.4%) did not know or refused to answer

### Content coding of the two open-ended questions of SDM-Q-9

Completion rate was 100% for all items of SDM-Q-9, as all questions were mandatory in the online survey. Median (Q1–Q3) completion time of SDM-Q-9 including the two open-ended questions was 2.17 (1.45–3.10) min. Coding of the two open-ended questions of SDM-Q-9 is demonstrated in Table 2. Overall, 20 groups of medical specialties and an ‘unspecified’ category were developed to classify the text responses with regard to the reason for consultation. A total of 586 problems were reported by the respondents. The most frequent reasons for consultation were musculoskeletal problems (*n* = 97; 18.1%), followed by cardiovascular problems (*n* = 80; 14.9%) and infection (*n* = 63; 11.7%). With regard to the type of decision, a total of 602 decisions were reported by the respondents, the most common of which were treatment (*n* = 424; 79.0%), diagnosis or screening test (*n* = 77; 14.3%) and referral (*n* = 45; 8.4%).

**Table 2:** Content coding for the two open-ended questions of SDM-Q-9

SDM-Q-9 question	*n* (%)
Reason for the visit (complaint/problem/illness)^a^	
Musculoskeletal	97 (18.1%)
Cardiovascular	80 (14.9%)
Infection	63 (11.7%)
Gastrointestinal	48 (8.9%)
Metabolic (incl. diabetes)	41 (7.6%)
Neurological	35 (6.5%)
Urinary (incl. kidney diseases)	20 (3.7%)
Dermatological	18 (3.4%)
Gynaecological	18 (3.4%)
Pulmonary	17 (3.2%)
Endocrinological	17 (3.2%)
Ophtalmological	16 (3.0%)
Oncological	14 (2.6%)
Psychiatric	14 (2.6%)
Reproductive	13 (2.4%)
Traumatological	13 (2.4%)
Prevention	10 (1.9%)
Allergological/immunological	6 (1.1%)
Otolaryngological	6 (1.1%)
Occupational	3 (0.6%)
Unspecified^b^	37 (6.9%)
Type of decision made^c^	
Treatment	424 (79.0%)
Diagnosis or screening test	77 (14.3%)
Referral	45 (8.4%)
Lifestyle	43 (8.0%)
Monitoring/follow-up	13 (2.4%)

^a^A total of 586 problems were reported by 537 respondents. Altogether 492 (91.6%), 41 (7.6%) and 4 (0.7%) respondents reported 1, 2 and 3 separate health problems, respectively

^b^Responses that cannot be clearly classified into the existing groups (e.g. ‘bleeding’)

^c^A total of 602 decisions were reported by 537 respondents. There were 465 (86.6%) respondents with 1 decision and 72 (13.4%) indicating 2 types of decision made

### Descriptive statistics of the nine items of SDM-Q-9

**Fig. 2:**
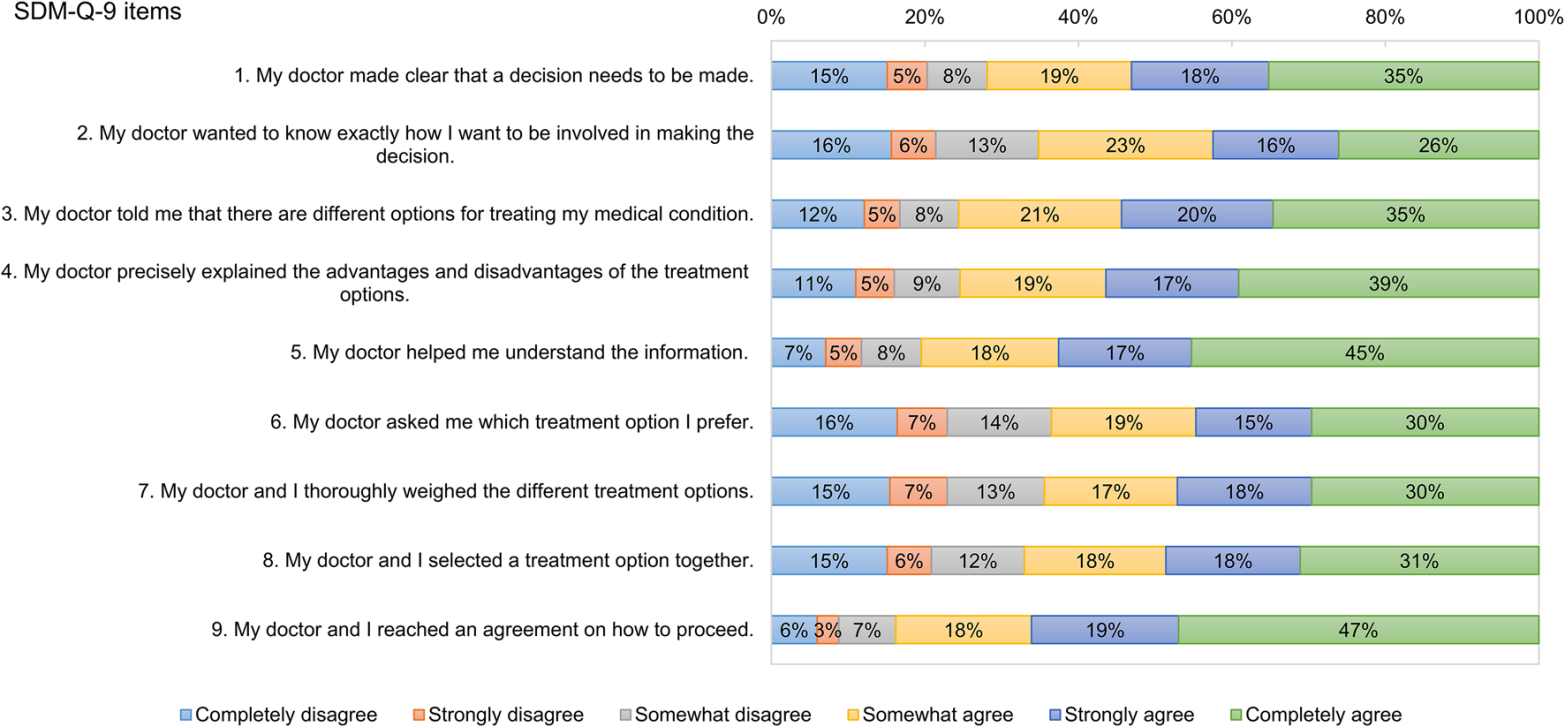
Distribution of responses on the nine items of SDM-Q-9. *SDM-Q-9* 9-item Shared Decision Making Questionnaire

In the total sample, mean (SD) SDM-Q-9 total score was 66.1 (26.7). No ceiling (12.3%) or floor effects (2.2%) were detected for total scores. Participants having a specialised care consultation indicated higher SDM-Q-9 total scores (mean 67.9 vs. 63.4, *p* = 0.0564). Figure 2 reports the frequency distribution of each item of SDM-Q-9. Overall, 117 (21.8%) respondents were ‘straight-liners’ selecting the same response option for each item. The majority of straight-liners marked positive responses: ‘completely agree’ (35.2%), ‘somewhat agree’ (18.8%) and ‘strongly agree’ (17.9%).

Item 9 displayed the highest ceiling effect and the lowest floor effect (46.9% and 6.0%), followed by item 5 (45.3% and 7.1%) and item 4 (39.1% and 11.0%). A higher overall ceiling effect (*p* = 0.0942) parallel to a lower floor effect (*p* = 0.0303) was observed for the specialised care subsample (Table 3).

**Table 3:** Item characteristics of SDM-Q-9

Items	Ceiling effect (*n*, %)	Floor effect (*n*, %)	Difficulty^b^ (mean, SD)	Discrimination (corrected item-total correlation)	Internal consistency (Cronbach’s α if the item deleted
Total sample (*n* = 537)^a^					
Item 1	189 (35.2%)	81 (15.1%)	3.25 (1.78)	0.470	0.933
Item 2	140 (26.1%)	84 (15.6%)	2.97 (1.73)	0.715	0.917
Item 3	186 (34.6%)	65 (12.1%)	3.36 (1.68)	0.740	0.915
Item 4	210 (39.1%)	59 (11.0%)	3.44 (1.68)	0.820	0.910
Item 5	243 (45.3%)	38 (7.1%)	3.69 (1.55)	0.751	0.915
Item 6	159 (29.6%)	88 (16.4%)	2.99 (1.79)	0.794	0.912
Item 7	159 (29.6%)	83 (15.5%)	3.03 (1.78)	0.847	0.908
Item 8	167 (31.1%)	81 (15.1%)	3.11 (1.77)	0.783	0.913
Item 9	252 (46.9%)	32 (6.0%)	3.82 (1.46)	0.656	0.921
Scale	66 (12.3%)	12 (2.2%)	3.29 (1.34)	–	0.925
Primary care sample (*n* = 211)					
Item 1	62 (29.4%)	35 (16.6%)	3.10 (1.78)	0.432	0.938
Item 2	51 (24.2%)	34 (16.1%)	2.88 (1.72)	0.684	0.922
Item 3	61 (28.9%)	29 (13.7%)	3.24 (1.68)	0.774	0.917
Item 4	69 (32.7%)	31 (14.7%)	3.22 (1.75)	0.845	0.912
Item 5	85 (40.3%)	18 (8.5%)	3.55 (1.60)	0.773	0.917
Item 6	60 (28.4%)	36 (17.1%)	2.93 (1.79)	0.796	0.915
Item 7	55 (26.1%)	36 (17.1%)	2.93 (1.80)	0.850	0.911
Item 8	61 (28.9%)	31 (14.7%)	3.07 (1.76)	0.783	0.916
Item 9	84 (39.8%)	15 (7.1%)	3.61 (1.51)	0.706	0.921
Scale	20 (9.5%)	8 (3.8%)	3.17 (1.36)	–	0.927
Specialised care sample (*n* = 320)					
Item 1	127 (39.7%)	43 (13.4%)	3.38 (1.76)	0.482	0.929
Item 2	88 (27.5%)	47 (14.7%)	3.05 (1.72)	0.732	0.912
Item 3	124 (38.8%)	35 (10.9%)	3.44 (1.68)	0.717	0.913
Item 4	141 (44.1%)	27 (8.4%)	3.60 (1.62)	0.799	0.908
Item 5	157 (49.1%)	19 (5.9%)	3.81 (1.50)	0.728	0.913
Item 6	99 (30.9%)	50 (15.6%)	3.05 (1.79)	0.790	0.908
Item 7	103 (32.2%)	46 (14.4%)	3.10 (1.78)	0.847	0.904
Item 8	106 (33.1%)	48 (15.0%)	3.16 (1.77)	0.785	0.908
Item 9	165 (51.6%)	16 (5.0%)	3.97 (1.39)	0.621	0.919
Scale	46 (14.4%)	3 (0.9%)	3.39 (1.31)	–	0.922
Primary vs. specialised	Fisher’s exact test *p* = 0.1072	Fisher’s exact test *p* = 0.0303	Student’s t test = − 1.91 (*df* = 529), *p* = 0.0564	–	Feldt’s test *χ*^2^ = 0.22 (*df* = 1), *p* = 0.6382

^a^Data about the level of care were indicated as ‘other’ for *n* = 6 respondents

^b^Difficulty is measured on a 0–5 scale

### Item difficulty, discrimination and internal consistency

Item characteristics including difficulty, discrimination and internal consistency reliability are presented in Table 3. All item difficulty values were above the midpoint of 2.5 with the highest means observed for item 8 and item 5, while the lowest for items 2 and 6. Compared to primary care, specialised care consultations were evaluated as being less difficult (mean item difficulty 3.39 vs. 3.17, *p* = 0.0564). In the total sample, corrected item-total correlations did not meet the threshold of > 0.70 for items 1 and 9. The overall internal consistency reliability was excellent (Cronbach’s *α* = 0.925). With respect to Cronbach’s *α*, there was no statistically significant difference between primary and specialised care (0.927 vs. 0.922; *p* = 0.6382).

### Construct validity

#### Exploratory factor analysis (EFA)

EFA resulted in one main factor with an eigenvalue > 1 for all three samples studied. The scree plot also indicated that one factor was responsible for the majority (63.49%) of the variance in SDM-Q-9. The explained variances were very similar for primary and specialised care (64.61% vs. 62.45%). The KMO measure verified an excellent sampling adequacy (0.910 for the total sample, 0.907 for primary care and 0.898 for specialised care). The Bartlett’s test for sphericity confirmed the statistical relevance of the models (*p* < 0.0001).

Table 4 shows the factor loadings and communalities for all items. For the total sample, individual loadings were high (i.e. ≥ 0.7) for all but one items. Item 1 produced a mediocre item loading of 0.540. In line with this, communalities of item 1 fell behind the required value of > 0.5. A very similar pattern was identified for primary care, whereas for specialised care communalities of items 1 and 9 were below the threshold. Regarding inter-item correlations, all values were below the recommended upper limit of 0.85 (total sample 0.311–0.826, primary care 0.259–0.839 and specialised care 0.333–0.821) indicating that there was no overlap between items.

**Table 4:** Results of the exploratory factor analysis (EFA)

Items	Total sample (*n* = 537)^a^		Primary care (*n* = 211)		Specialised care (*n* = 320)	
	Factor loadings	Communalities (*h*^2^)	Factor loadings	Communalities (*h*^2^)	Factor loadings	Communalities (*h*^2^)
Item 1	0.540	0.292	0.497	0.247	0.554	0.307
Item 2	0.766	0.588	0.739	0.546	0.782	0.611
Item 3	0.802	0.643	0.827	0.683	0.784	0.615
Item 4	0.872	0.760	0.891	0.793	0.855	0.732
Item 5	0.813	0.661	0.826	0.683	0.797	0.635
Item 6	0.851	0.725	0.854	0.730	0.849	0.721
Item 7	0.895	0.801	0.901	0.812	0.893	0.797
Item 8	0.845	0.714	0.848	0.719	0.846	0.716
Item 9	0.730	0.533	0.775	0.600	0.698	0.487
KMO	0.910		0.907		0.898	
Bartlett’s test	*χ*^2^ = 3560.87 (*df* = 36), *p* < 0.0001		*χ*^2^ = 1476.26 (*df* = 36), *p* < 0.0001		*χ*^2^ = 2067.34 (*df* = 36), *p* < 0.0001	

*KMO* Kaiser–Meyer–Olkin measure

^a^Data about the level of care were indicated as ‘other’ for *n* = 6 respondents

#### Confirmatory factor analysis

Table 5 presents the results of the CFA. The overall performance of the four models was very similar. Almost every model met the cut-off criteria of *χ*^2^, CFI and SRMR, but none achieved an acceptable RMSEA value. Model 1 (all nine items included) demonstrated a more or less acceptable performance with CFI = 0.899, RMSEA = 0.158 and SRMR = 0.052. For the total sample as well as the two subsamples, the best performing model in terms of fit indices was model 4 whereby both items 1 and 9 were excluded.

**Table 5:** Results of the confirmatory factor analysis (CFA)

No.	Model	Estimator	*df*	*χ* ^2^	CFI	RMSEA	SRMR
Total sample (*n* = 537)							
1	One-factor model including all items	ML	27	387.39*	0.899	0.158	**0.052**
		SB		204.53*	**0.916**	0.111	N/A
2	One-factor model excluding item 1	ML	20	260.28*	**0.928**	0.150	**0.039**
		SB		129.89*	**0.943**	0.101	N/A
3	One-factor model excluding item 9	ML	20	324.06*	**0.905**	0.168	**0.053**
		SB		168.87*	**0.923**	0.118	N/A
4	One-factor model excluding items 1 and 9	ML	14	195.81*	**0.939**	0.156	**0.035**
		SB		94.79*	**0.954**	0.104	N/A
Primary care (*n* = 211)							
1	One-factor model including all items	ML	27	168.44*	**0.904**	0.158	**0.054**
		SB		82.67*	**0.932**	0.099	N/A
2	One-factor model excluding item 1	ML	20	119.11*	**0.929**	0.153	**0.038**
		SB		56.07*	**0.953**	0.092	N/A
3	One-factor model excluding item 9	ML	20	147.03*	**0.903**	0.174	**0.058**
		SB		75.06*	**0.929**	0.114	N/A
4	One-factor model excluding items 1 and 9	ML	14	97.29*	**0.932**	0.168	**0.038**
		SB		47.64*	**0.954**	0.107	N/A
Specialised care (*n* = 320)							
1	One-factor model including all items	ML	27	266.40*	0.884	0.166	**0.056**
		SB		152.49*	**0.904**	0.121	N/A
2	One-factor model excluding item 1	ML	20	181.28*	**0.916**	0.159	**0.046**
		SB		96.08*	**0.934**	0.109	N/A
3	One-factor model excluding item 9	ML	20	208.03*	0.899	0.171	**0.055**
		SB		113.01*	**0.920**	0.121	N/A
4	One-factor model excluding items 1 and 9	ML	14	123.74*	**0.936**	0.157	**0.039**
		SB		60.24*	**0.955**	0.102	N/A

Recommended values: CFI > 0.90, RMSEA and SRMR ≤ 0.08. Values meeting the cut-off criteria are indicated in bold

*CFI* comparative fit index, *ML* maximum likelihood, *N/A* not applicable, *RMSEA* root mean square error of approximation, *SB* Satorra-Bentler, *SRMR* standardized root mean square residual

*p < 0.0001

### Known-groups validity

**Fig. 3:**
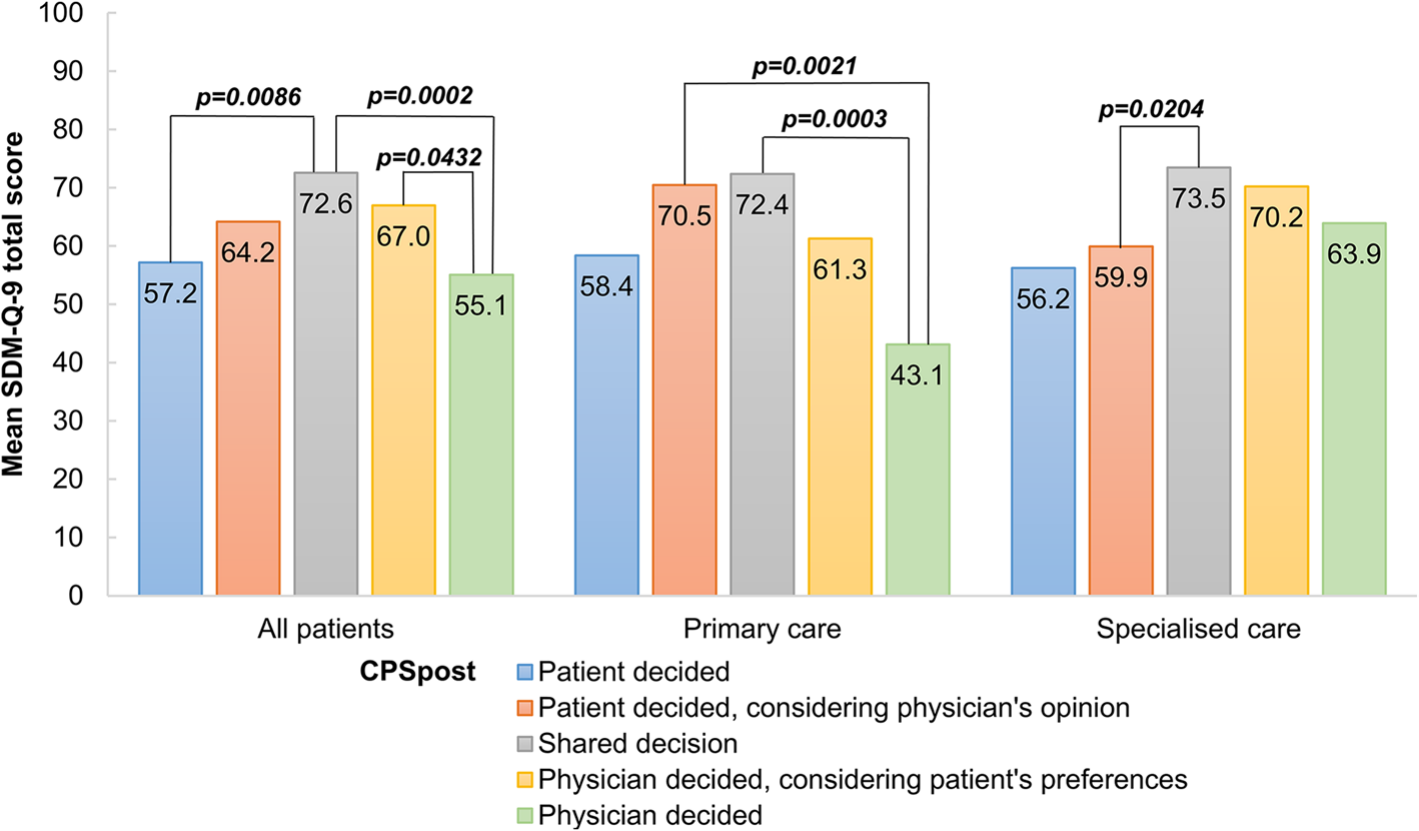
Known-groups validity: mean total SDM-Q-9 scores by CPS_post_ category. Note that *p* values indicate Games-Howell post hoc test. *CPS*_*post*_ Control Preferences Scale post, *SDM-Q-9* 9-item Shared Decision Making Questionnaire

Figure 3 shows the mean SDM-Q-9 total scores according to the five CPS_post_ categories. As expected, the ANOVA found significant differences in SDM-Q-9 total scores across CPS_post_ categories (total sample and primary care *p* < 0.0001, specialised care *p* = 0.0021). In the total sample, ‘Shared decision’ was associated with significantly higher mean SDM-Q-9 total score (72.6) compared to both ‘Physician decided’ (55.1), ‘Physician decided considering patient’s preferences’ (67.0) and ‘Patient decided’ (57.2) categories (*p* < 0.05). The difference between the ‘Shared decision’ and ‘Patient decided considering physician’s opinion’ (64.2, *p* = 0.0840) categories also showed a trend towards statistical significance.

In the primary care sample, mean SDM-Q-9 total scores of ‘Physician decided’ category (43.1) were significantly lower compared to both the ‘Shared decision’ (72.4) and ‘Patient decided considering physician’s opinion’ (70.5). In the specialised care subsample, mean SDM-Q-9 total score of the ‘Shared decision’ category (73.5) was significantly higher than that of ‘Patient decided considering physician’s opinion’ (59.9).

## Discussion

In this study a Hungarian version of the SDM-Q-9 questionnaire was developed and psychometrically tested. The overall data quality was reasonably acceptable; however, over one-fifth of the population provided response patterns. No ceiling or floor effects were observed for SDM-Q-9 total scores. In accordance with former validation studies, an appropriate difficulty was observed for all items. The results regarding internal consistency reliability (Cronbach’s *α* = 0.925) are comparable to the first psychometric testing of the original German questionnaire (0.938) and that of the Danish (0.94), Dutch (0.88), Romanian (0.95) and Spanish (0.885) versions [14, 30, 31, 33, 34].

Results of the factor analyses supported the single-factor construct of the original German SDM-Q-9 [14]. The one-structure model explained 63.5% of the variance of SDM-Q-9 in Hungary versus 62.4% in Germany. In contrast the Dutch, Romanian and Spanish versions revealed a two-component structure of the instrument [30, 31, 33]. In our one-factor model, supporting the results of the discrimination and item-level reliability, items 1 (‘My doctor made clear that a decision needs to be made’) and 9 (‘My doctor and I reached an agreement on how to proceed’) contributed the least to the variance. Thus, we decided to test the effect of eliminating these items in a CFA. It was found that by removing these items, all fit indices slightly improved. Nonetheless, to be consistent with all other language versions of SDM-Q-9, it was decided to keep all nine items in the Hungarian version.

The SDM-Q-9 demonstrated an excellent known-groups validity in distinguishing between groups of patients based on their CPS_post_ categories. Perception of a more autonomous role of the respondent on CPS_post_ was associated with a higher mean SDM-Q-9 score corresponding to a higher involvement in the decision made. The differences were particularly marked between the ‘Shared decision’ (72.6), ‘Patient decided’ (57.2) and ‘Physician decided’ (55.1) categories. Known-groups validity has earlier been analysed by the same method in the Dutch validation study that enrolled both primary and specialised care patients. In their study mean SDM-Q-9 total scores across the five CPS_post_ groups were similar to those found in our study: ‘Patient decided’ (73.1), ‘Patient decided, considering physician’s opinion (80.1), ‘Shared decision’ (81.1), ‘Physician decided, considering patient’s preferences (64.9) and ‘Physician decided’ (39.4) [30].

The inter-country variations in psychometrics of the SDM-Q-9 may be attributable to the differences across studies in terms of patient characteristics (diagnosis, mean age, decisions assessed), levels of care (primary, specialised or both), data collection methods (paper-based or online), nuances in language versions of the questionnaire and cultural variations in patient–physician relationships. Taking as a whole, measurement properties of the Hungarian SDM-Q-9 are very close to those of the original German version.

The large sample size of the study allowed to explore the potential differences in properties of SDM-Q-9 between primary and specialised care subsamples. Only small variations were found between the two settings, and the overall good performance of the measure was true for both subsamples. The questionnaire showed a decreased ceiling effect and improved internal consistency and factor structure in primary care, whereas discrimination and item difficulty were slightly better for specialised care. Interestingly, compared to specialised care, much lower SDM-Q-9 total scores were found in primary care for the two categories referring to a passive patient role. This may imply that patients have different expectations regarding the SDM process in primary and specialised care. It seems that a greater involvement of physicians may be acceptable in specialised care settings.

The first strength of our study was using a large nationally representative sample of the general population for the validation. This enabled to reach a variety of groups of patients with different diagnoses including acute and chronic conditions. To our knowledge, we were the first to compare the validity and reliability of SDM-Q-9 in primary and specialised care settings. Furthermore, this is the first study in the literature evaluating pattern answering and completion time of the SDM-Q-9.

Our study has some limitations. First, recall bias could have arisen as participants were asked to retrospectively recall health-related decisions using a 6-month time frame. It is very likely, however, that the time between the decision and the completion of the survey was much shorter, especially when one takes into account the proportion of respondents with chronic diseases in the sample. Second, as opposed to previous validation studies, the assessment of the acceptance rates of the questionnaire items was not possible, as all questions of SDM-Q-9 were mandatory in the online survey.

In conclusion, the present study is the first national survey on SDM practices in Hungary. The overall good measurement properties of the Hungarian SDM-Q-9 make the questionnaire suitable for use both in primary and specialised care settings. The results may facilitate the understanding of the SDM process in the Hungarian context and aspire to ground health policies targeting the implementation of SDM practices in Hungary.
